# Hookahs: Hot and Hazardous

**DOI:** 10.1289/ehp.119-a339

**Published:** 2011-08-01

**Authors:** Carol Potera

**Affiliations:** Carol Potera, based in Montana, has written for *EHP* since 1996. She also writes for *Microbe*, *Genetic Engineering News*, and the *American Journal of Nursing*.

Hookah cafes are an increasingly popular venue for socializing. In addition to beverages, appetizers, and desserts, habitués can order different flavors of tobacco that they smoke through waterpipes. Many patrons of hookah cafes believe smoking a waterpipe is safer than smoking cigarettes—an unsubstantiated belief “as old as the waterpipe itself,” according to the World Health Organization.^1^ A new field trial shows that carbon monoxide (CO) levels were 3 times higher in people visiting hookah cafes than in people who visited traditional bars.^2^

Tracey Barnett, a social and behavioral scientist at the University of Florida, Gainesville, and colleagues measured CO levels of 173 patrons leaving three local hookah cafes and 198 patrons leaving five traditional bars that allow smoking. Hookah cafe patrons had an average CO level of 30.8 ppm compared with 8.9 ppm for traditional bar patrons. Even hookah cafe patrons who did not smoke from the waterpipe had average elevated CO levels of 11.5 ppm, similar to cigarette smokers.^2^

The Occupational Safety and Health Administration established a cutoff of 50 ppm for CO exposure over an 8-hour period;^3^ 18% of hookah cafe patrons had CO levels exceeding this level, and 5% tested above 90 ppm.^2^ Symptoms of CO poisoning such as lightheadedness and nausea start at about 70 ppm.^4^ Some hookah smokers claim they experience a “high,” but “they’re probably in the early stages of CO poisoning,” Barnett says. Emergency rooms have reported visits for CO poisoning after hookah smoking.^5^^,^^6^^,^^7^

Hookah smoke contains toxicants not only from burning tobacco but also from the charcoal used to heat the tobacco in the pipe’s bowl, including CO, heavy metals, and polycyclic aromatic hydrocarbons.^8^ Shared hookahs also can raise the risk for communicable diseases.^9^ The water in a waterpipe does absorb some nicotine, so hookah smokers may inhale more smoke seeking a satisfying amount of the drug.^1^ A hookah session typically lasts 20–80 minutes, and the number and depth of puffs taken means a patron may inhale the smoke equivalent of 100 or more cigarettes.**^1^**

Hookah cafes are popular in university towns and large cities. By one 2005 estimate, up to 20% of some U.S. populations of young adults engage in hookah smoking.^10^ Norman Edelman, chief medical officer at the American Lung Association, says his organization is working with states to pass laws to ban hookah smoking. “People realize more and more that this is a dangerous practice,” Edelman says.
